# Progress in the Development of Eukaryotic Elongation Factor 2 Kinase (eEF2K) Natural Product and Synthetic Small Molecule Inhibitors for Cancer Chemotherapy

**DOI:** 10.3390/ijms22052408

**Published:** 2021-02-27

**Authors:** Bin Zhang, Jiamei Zou, Qiting Zhang, Ze Wang, Ning Wang, Shan He, Yufen Zhao, C. Benjamin Naman

**Affiliations:** 1 Li Dak Sum Yip Yio Chin Kenneth Li Marine Biopharmaceutical Research Center, College of Food and Pharmaceutical Sciences, Ningbo University, Ningbo 315800, China; zhangbin1@nbu.edu.cn (B.Z.); 186003328@nbu.edu.cn (J.Z.); 2011085073@nbu.edu.cn (Z.W.); heshan@nbu.edu.cn (S.H.); 2 Institute of Drug Discovery Technology, Ningbo University, Ningbo 315211, China; 1811075025@nbu.edu.cn (Q.Z.); zhaoyufen@nbu.edu.cn (Y.Z.)

**Keywords:** eEF2K, protein kinase, enzyme inhibitors, natural products, small molecules, cancer therapy, medicinal chemistry

## Abstract

Eukaryotic elongation factor 2 kinase (eEF2K or Ca^2+^/calmodulin-dependent protein kinase, CAMKIII) is a new member of an atypical α-kinase family different from conventional protein kinases that is now considered as a potential target for the treatment of cancer. This protein regulates the phosphorylation of eukaryotic elongation factor 2 (eEF2) to restrain activity and inhibit the elongation stage of protein synthesis. Mounting evidence shows that eEF2K regulates the cell cycle, autophagy, apoptosis, angiogenesis, invasion, and metastasis in several types of cancers. The expression of eEF2K promotes survival of cancer cells, and the level of this protein is increased in many cancer cells to adapt them to the microenvironment conditions including hypoxia, nutrient depletion, and acidosis. The physiological function of eEF2K and its role in the development and progression of cancer are here reviewed in detail. In addition, a summary of progress for in vitro eEF2K inhibitors from anti-cancer drug discovery research in recent years, along with their structure–activity relationships (SARs) and synthetic routes or natural sources, is also described. Special attention is given to those inhibitors that have been already validated in vivo, with the overall aim to provide reference context for the further development of new first-in-class anti-cancer drugs that target eEF2K.

## 1. Introduction

Targeted therapy is an important strategy for cancer treatment, and this has been well applied in actual clinical applications [1]. At present, most clinically used targeted cancer drugs are inhibitors of tyrosine kinases [1,2]. However, even when these drugs have exceptional efficacy initially, the later emergence of drug resistance limits their usefulness [2]. Finding new drug targets and developing new targeted anticancer agents have accordingly become important aspects of drug discovery and development.

Eukaryotic elongation factor 2 kinase (eEF2K) is the first α-kinase to have been discovered [3]. The activity of eEF2K depends on calcium ions and calmodulin (CaM) [4]. Therefore, eEF2K has also been called CaM-dependent protein kinase III or CAMKIII, and it appears this way in some literature reports [5]. The “α-kinase” is an atypical protein kinase family, and while these often have similar ATP binding pockets to typical protein kinase family members, they are differentiated by having a translocated conserved region with an alternate sequence, GXGXXG [6]. Because of this, drugs that target eEF2K are less likely to modulate typical kinases and should ideally not have or create cross-resistance with traditional kinase drugs. Increasing evidence shows that eEF2K is highly expressed in a variety of tumor tissues and that it is related to the development and prognosis of several kinds of malignancies such as breast cancer, ovarian cancer, colon cancer, glioma, medulloblastoma, hepatocellular carcinoma, and prostate cancer [7,8,9,10,11,12]. In addition, eEF2K can also participate in the regulation of the tumor cell cycle, proliferation, autophagy, apoptosis, angiogenesis, invasion, and metastasis, among other processes [13,14,15]. For all of these reasons, eEF2K is a potential therapeutic target for anticancer drug development.

Recently, with the deepening research on the function of eEF2K, increased attention has turned to the development of eEF2K inhibitors. However, relatively fewer reports have disclosed the development of eEF2K inhibitors compared with studies on the physiological functions and pathological effects of eEF2K. While the molecules summarized in this review have been reported as eEF2K inhibitors, the chemical structures are very different, and no predominant pharmacophore or active scaffold has emerged. Herein, the physiological functions of eEF2K are introduced and its various regulatory effects on tumors are discussed. Furthermore, a summary of recent research progress on eEF2K inhibitors for cancer chemotherapy is presented along with the detailed structure–activity relationships (SARs) and synthetic routes or natural sources of existing eEF2K inhibitors.

## 2. Physiological Function of eEF2K

Cellular and organismal survival requires the continuous maintenance of protein synthesis, categorizing proteins as essential for function. During protein synthesis, the control of elongation regulates mRNA translation and adapts cells to changes in nutrients, energy, and oxygen. The protein eukaryotic elongation factor 2 (eEF2) plays a key role in protein elongation and is the only substrate of eEF2K known to date [16]. As a member of the GTP-binding translation elongation factor family, eEF2 promotes GTP-dependent ribosome translocation [17]. During elongation, eEF2 promotes the movement of peptidyl-tRNAs from the A site to the P site of the ribosome. In eukaryotic cells, eEF2 can be completely inactivated via phosphorylation by eEF2K [18]. Currently, eEF2K is the only α-kinase with activity dependent on Ca^2+^ ions [19]. In vivo, Ca^2+^ ions bind to calmodulin with high affinity and activate the eEF2K kinase domain, and this rapidly triggers the autophosphorylation of eEF2K at Thr384, leading to kinase conformational changes that form a binding pocket [13]. Active eEF2K will phosphorylate eEF2 at Thr56, thereby inhibiting the activity of eEF2 and ultimately preventing the extension of polypeptide chains in the process of protein synthesis [20]. What is known about the structure of eEF2K and the specific regulation of the extension process has been reviewed in detail by Proud [5], as well as more recently by Karakas and Ozpolat [13], and will not be elaborated here.

## 3. The Role of eEF2K in Cancer

It has been shown that eEF2K is overexpressed and regulates tumor progression in several types of malignancies, including breast cancer, glioma cancer, pancreatic cancer, lung cancer, neuroblastoma, and colorectal cancer [8,21,22]. Previous studies have found that eEF2K is associated with tumor proliferation and survival, tumorigenesis, invasion, drug resistance, and poor prognosis [23,24]. For instance, microRNA 603 inhibits tumor formation in triple-negative breast cancer by the targeted inhibition of eEF2K [25]. It was also reported that eEF2K promotes the proliferation of ovarian cancer cells and that its expression is positively correlated with poor prognosis [24]. In hepatocellular carcinoma, eEF2K promotes angiogenesis through PI3K/Akt and STAT3 signaling [11]. Similarly, eEF2K is positively correlated with lung cancer proliferation, invasion and metastasis, and poor prognosis [26]. These early data indicate the importance of eEF2K in cancers, and suggest that it is a potential new target for cancer chemotherapeutic treatments.

### 3.1. eEF2K Helps Tumor Cells to Cope with Harsh Environments

**Figure 1:**
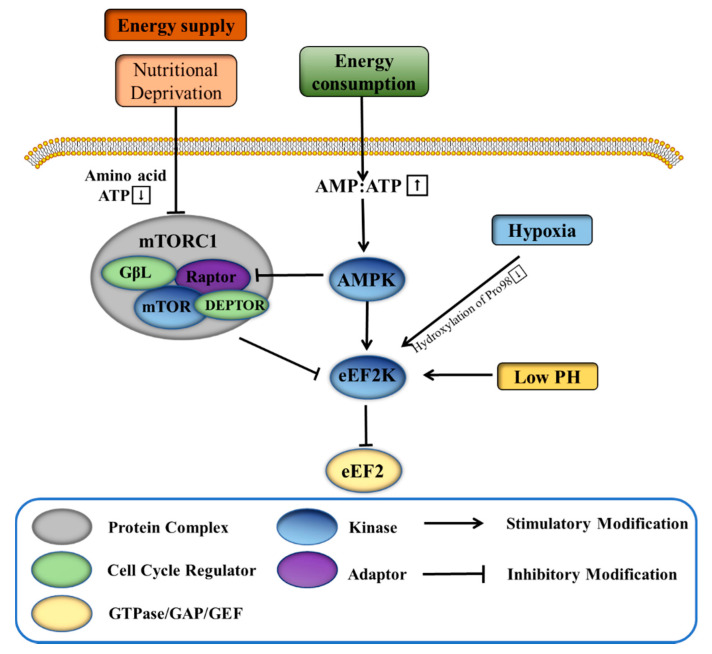
The effects that some typical tumor microenvironmental conditions have on eEF2K.

The rapid proliferation of cancer cells in tumors requires a high amount of energy, in part due to greatly upregulated protein synthesis. The process leads to tumors creating their own harsh microenvironments that have, for example, reduced availability of nutrients, low pH, and insufficient oxygen. Further adaptation of the tumor cells, including by increasing expression of eEF2K, helps to regulate the synthesis of proteins in the harsh environment and be protected for continued proliferation. Under conditions of nutritional deprivation, tumor cells with high eEF2K expression can continue to survive, while those with low eEF2K expression have been shown to die [27]. When intracellular nutrition is insufficient, the content of ATP decreases, and AMP or ADP increases to activate AMP-activated protein kinase (AMPK). After activation, AMPK can induce the phosphorylation of eEF2K at Ser398 or Ser491 to thereby inhibit the function of eEF2 (Figure 1). This ultimately reduces the rate and energy consumption of intracellular proliferation and protein synthesis and promotes energy production processes such as glucose metabolism and fatty acid oxidation. The mammalian target of rapamycin complex 1 (mTORC1) is another energy-related protein that has been found to downregulate eEF2K [28]. Activated mTORC1 inhibits the activation of eEF2K by inducing its phosphorylation at a variety of residues, including Ser70, Ser78, Ser359, Ser366, Ser392, Ser396, and Ser470 [29,30]. Regarding its regulation, mTORC1 is stimulated by amino acids, hormones, growth factors, and cellular nutrients [4,28]. Under nutritional deprivation, the activity of mTORC1 is inhibited, partially alleviating its phosphorylation and inhibitory effect on eEF2K. At the same time, activated AMPK has been shown to further indirectly inhibit the activity of mTORC1 [31]. In addition, mTORC1-mediated inhibition of eEF2K is essential for proliferation of adenomatous polyposis coli (APC)-deficient cells. Rapamycin targets eEF2 indirectly through the mTORC1-S6K-eEF2K pathway, and treatment of APC-deficient adenomas with rapamycin induces tumor cell growth arrest and differentiation [32].

The rapid proliferation of tumor cells also requires a large amount of oxygen, which frequently is overdrawn enough to induce a hypoxic environment. It has been found that hypoxia inhibits protein synthesis in breast cancer cells in part through 4E-BP1 and the eEF2K pathway, controlled by mTOR [33]. Additionally, eEF2K is activated and induces eEF2 phosphorylation during hypoxia independent of AMPK and mTORC1 signaling [34]. The eEF2K residue Pro98 is a generally conserved linker between the calmodulin binding domain and the catalytic domain, and when it is hydroxylated, the binding of calmodulin to eEF2K is reduced and the activity of eEF2K is significantly limited. Under normoxia, proline hydroxylase catalyzes the hydroxylation of Pro98 of eEF2K, thus inhibiting eEF2K activity. However, when the cells are hypoxic, the activity of proline hydroxylase is inhibited, thereby releasing the normal inhibition of eEF2K (Figure 1) [34]. Normal cells rely on mitochondrial oxidative phosphorylation to produce energy, while tumor cells mainly generate energy through glycolysis under hypoxic/normoxic conditions (Warburg effect) [35]. The upregulation of eEF2K accelerates glycolysis to promote human breast cancer cells in development and progression. eEF2K inhibits protein phosphatase 2A-A (PP2A-A) synthesis, thereby interfering with its promotion of c-Myc ubiquitin-proteasome degradation, and finally activates the transcription of pyruvate kinase M2 subtype (PKM2) to promote glycolysis [36].

Low pH is frequently a major feature of tumor microenvironments. In tumor cells, unrestricted glycolysis leads to a large accumulation of lactic acid, thereby acidifying the local environment [37,38]. Under acidic conditions, or low pH, overexpression of eEF2K inhibits protein synthesis. At neutral pH, by contrast, overexpression of eEF2K does not affect protein synthesis, indicating the activation of eEF2K in acidic conditions (Figure 1) [39]. However, the activation of eEF2K is independent of the activity of mTORC1. It is understood that the affinity of eEF2K to CaM is enhanced at acidic pH, and the histidine residue (H108) in CaM is essential for the activation of eEF2K [40].

Overall, the activity of eEF2K is known to change with the conditions of the tumor microenvironment (e.g., energy deficiency, hypoxia, low pH), thereby regulating the process of tumor protein synthesis and ultimately protecting the survival of tumor cells in otherwise harsh conditions.

### 3.2. eEF2K Inhibits Cell Apoptosis

**Figure 2:**
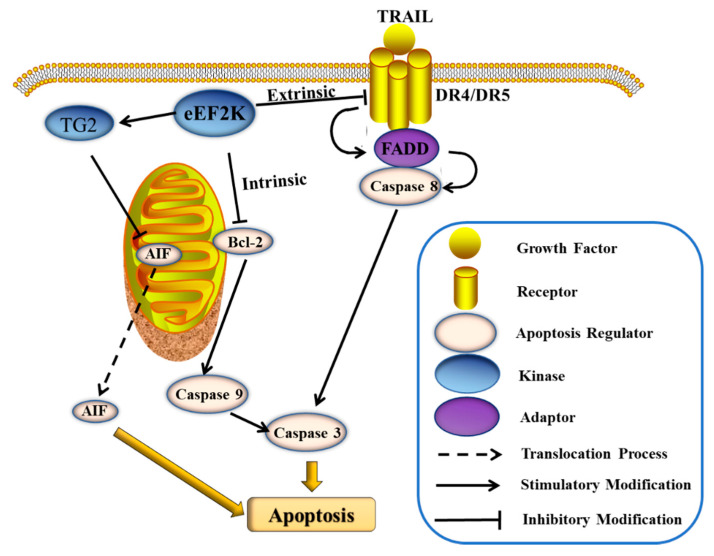
Some pathway effects of eEF2K on apoptosis in tumor cells.

Cell apoptosis is typically dysregulated in cancers, which leads to rampant proliferation. Accordingly, inducing apoptosis in cancer cells is an important mechanism of anti-tumor drugs. The expression of eEF2K can inhibit apoptosis and promote cancer cell survival [4]. The suppression of this mechanism would enable eEF2K to be used mechanistically as a new drug target for single or multi-agent cancer chemotherapeutics. The caspases represent an important family of proteins that regulate cell apoptosis. Among them, caspase 8 and caspase 9 are the key regulatory proteins in extrinsic and intrinsic apoptotic pathways, respectively [41]. The cleavage of these caspases will eventually lead to the cleavage of caspase 3 and ultimately apoptosis of the cell [42]. Some eEF2K inhibitors have been shown to induce tumor cell apoptosis by these mediated extrinsic and/or intrinsic apoptosis pathways. The cleavage of caspase 8 that is induced by tumor necrosis factor (TNF) family proteins is an important aspect of the extrinsic apoptosis pathway [43]. The TNF-related apoptosis-inducing ligand (TRAIL) belongs to the TNF family. TRAIL can bind to the death receptors DR4 and DR5 to form the death-inducing signaling complex (DISC) and to upregulate Fas-associated protein with death domain (FADD), thereby inducing caspase-8-dependent apoptosis (Figure 2) [44]. Treatment of glioma cells with the eEF2K inhibitor, NH125 (**1**), showed the enhancement of TRAIL-induced apoptosis, and, with the increase of dosed NH125, the cleaved PARP and caspase 8 levels increased significantly [45]. Bcl-2 is another important family of proteins that regulate endogenous apoptosis [46]. The founding member protein Bcl-2 and Bcl-xL are anti-apoptotic proteins in the Bcl-2 family, and NH125 down-regulates the expression of Bcl-xL in glioma cells [45]. It was additionally shown that silencing eEF2K induces caspase-9 cleavage and Bcl-2 downregulation in breast cancer cells (Figure 2) [47]. Meanwhile, inhibiting eEF2K enhances the effect of doxorubicin in an orthotopic model of breast cancer [47]. Furthermore, eEF2K is highly expressed in pancreatic cancer (PaCa) and acts to inhibit apoptosis [14]. Treatment of PaCa cells with the natural product inhibitor of eEF2K, rottlerin (**29**), not only induces the collapse of mitochondrial potential causing intrinsic apoptosis, but also causes extrinsic apoptosis regulated by TRAIL and caspase 8 [14]. At the same time, rottlerin also effects the expression of TG2 (PKC-δ/tissue transglutaminase), which in turn activates apoptosis-inducing factor (AIF), and ultimately causes caspase-dependent apoptosis (Figure 2) [14].

### 3.3. eEF2K Regulates the Cell Cycle

**Figure 3:**
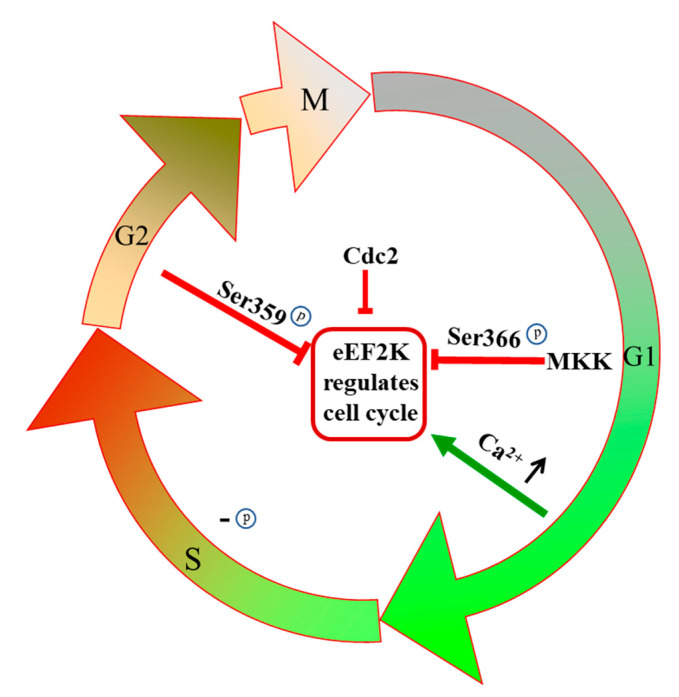
The regulatory effects of eEF2K on the cell cycle, especially prevalent in cancers.

The cell cycle is inextricably tied to protein synthesis, and thus the impact of eEF2K in elongation can be understandably expanded. For one thing, phosphorylation of eEF2K residues Ser359 and Ser366 leads to its inactivation, which accordingly regulates cell cycle progression [31]. The inhibition of eEF2K has been shown to arrest breast cancer cells at the G0/G1-S phase [48]. Conversely, eEF2K is inactivated during G1 cell growth, so eEF2 becomes active and promotes protein synthesis [48]. The Ser366 residue of eEF2K is a point of regulation by MKK and mTOR pathways, and when cells enter the G1 phase, Ser366 is phosphorylated rapidly in an MKK-dependent manner (Figure 3) [49]. During S phase DNA replication, eEF2K is slowly dephosphorylated, and eEF2 activity is eventually inhibited completely during G2 and mitosis (Figure 3) [48]. It is well-known that eEF2K is a calcium/calmodulin-dependent protein kinase, and calcium binding to CaM has an important influence on the process of mitosis [5,50]. In the G1/S transition, the intracellular Ca2+ concentration increases. Under environmental conditions of high calcium concentration, calmodulin can bind to eEF2K and activate it so that the cells enter the S phase (Figure 3) [51]. Another function of cellular calcium is to upregulate cAMP levels [52]. The cAMP in turn activates PKA, which can activate eEF2K by phosphorylation of the Ser500 residue [52,53]. The regulation of eEF2K on the G2/M phase is related to the phosphorylation at the Ser359 residue, and this leads to inhibition of eEF2K activity independent of Ca^2+^ concentration (Figure 3) [54]. It has been shown that eEF2K can also be regulated by cell cycle-related proteins to affect cell cycle progression. For example, human cyclin-dependent kinase 1 (CDC2) is regulated by mTORC1 and gets activated in the early stage of mitosis; it then inactivates eEF2K, and thus protein synthesis is carried out in mitotic cells (Figure 3) [55]. Cell cycle progression is closely related to proliferation in tumor cells, and chemical interference can accordingly inhibit proliferation and eventually lead to cell death [56]. The successful launch of the CDK4 and CDK6 targeting cell cycle inhibitor palbociclib, which was approved by the U.S. FDA in 2015, has encouraged further research on cell cycle-regulating anticancer agents for use as drugs [57]. Since eEF2K has a regulatory effect on multiple links in the cell cycle process, this represents an attractive new target for future cancer treatments.

### 3.4. eEF2K Regulates Cell Autophagy

Autophagy is a “self-consuming” program of cells that is used to remove damage and dysfunction or unnecessary proteins, and it is closely associated with human diseases such as cancer [58,59]. Under conditions of starvation or stress, excess cells may undergo autophagy so that the remaining cells can better survive [60,61]. Autophagy mechanistically works by lysosomes in cells degrading their own organelles and other macromolecules, and it is an important process for eukaryotes to carry out the turnover of intracellular substances [62,63]. As previously discussed, tumor cell proliferation often results in microenvironment nutritional starvation or stress conditions. Autophagy can protect cells from apoptosis and promotes tumor progression [62,64]. mTOR is an important regulator of autophagy and the upstream protein of eEF2K. Previous studies have demonstrated that eEF2K induces autophagy to protect cancer cells survival [65,66]. For example, it was found that during amino acid starvation and endoplasmic reticulum (ER) stress, eEF2K is activated to induce autophagy [67,68]. Inhibiting eEF2K-mediated autophagy has been reported to enhance the antitumor effect of MK-2206, an AKT inhibitor, on human nasopharyngeal carcinoma and human glioma cells [65,66]. The induction of autophagy is mediated via the TSC2/mTOR/S6 kinase/eEF-2 kinase pathway [65]. It was also found that silencing of eEF2K can inhibit autophagy through the mTORC1/p70S6K signaling pathway and increase the sensitivity of human glioma cells to 2-deoxy-d-glucose (2-DG) [69].

However, in contrast to the above outcome in human nasopharyngeal carcinoma and human glioma cells, it was found that silencing of eEF2K activity can induce autophagy to promote the proliferation of colon cancer cells but not enhance the anticancer effect of MK-2206 in human colon cancer cells [70]. The negative regulation of eEF2K on autophagy in colon cancer cells is dependent on the activation of the AMPK-ULK1 pathway [70]. Another experiment on human lung cancer cells showed that eEF2K protects cell survival under nutrient deprivation, but this effect is due to its inhibition of protein synthesis rather than regulation of autophagy [15]. Thus, the particular cancer cell type and specific mechanism of action being observed is important to consider.

### 3.5. eEF2K Promotes Tumor Angiogenesis, Metastasis, and Invasion

Angiogenesis, the growth of new blood vessels, provides tumors with more nutrients to promote their growth, and plays a key role in tumor proliferation, metastasis, and invasion [71,72,73]. Overexpression of eEF2K has been shown to promote angiogenesis, invasion, and metastasis in multiples types of tumors [74,75]. Inhibition of eEF2K expression can likewise prevent these tumor processes. For example, knockdown of eEF2K was found to prevent tumor progression and angiogenesis of hepatocellular carcinoma via the PI3K/Akt and STAT3 signaling pathway [11]. In triple-negative breast cancer (TNBC) cells, the proto-oncogene transcription factor forkhead box M1 (FOXM1) can regulate eEF2K and affect breast cancer cell migration and invasion, progression, and tumorigenesis [23]. The dual inhibitory effect of microRNA-34a on the FOXM1/eEF2K axis can regulate the growth and invasion of TNBC [76]. In addition, TNBC with mutations in PTEN and p53 is more sensitive to eEF2K inhibitors, and this effect is related to the AKT signaling pathway [8]. Similarly, it was found after knocking out eEF2K that the invasion and metastasis of lung cancer cells was inhibited [26]. Proud et al. found that this inhibitory effect may be related to integrin signaling proteins to control cell–cell/cell–extracellular matrix interactions and cell mobility [77].

## 4. Natural Product and Synthetic Small Molecule Inhibitors of eEF2K

Since eEF2K does not belong to a typical large family of kinases, and it is the only α-kinase that depends on Ca^2+^ and calmodulin, the structural dynamics of this protein have only recently been characterized [78]. Earlier studies, however, did find that blocking the function of eEF2K can effectively kill cancer cells without affecting normal cells [79]. Since the function of eEF2K is related to CaM, ATP, and eEF2, the corresponding binding sites are the key targets know for competitive inhibitors. Specific eEF2K inhibitors will ideally only affect the activity of the eEF2 protein to modulate elongation and cell cycle regulation, thus offering a new strategy for anticancer drug discovery and development. Many structurally distinct natural product and synthetic small molecule inhibitors of eEF2K have been reported to date (Table 1). However, due to the limited amount of existing research on eEF2K, no optimal pharmacophore or structure scaffold has been found for inhibiting this molecular target. Accordingly, the development of eEF2K inhibitors is still in the early preclinical stage. The previously reported eEF2K inhibitors are delineated according to whether they were discovered in single-target studies or as multi-target inhibitors, and each is described in the following sections.

### 4.1. Discovery and Development of Single Target eEF2K Inhibitors

#### 4.1.1. NH125

**Figure 4:**
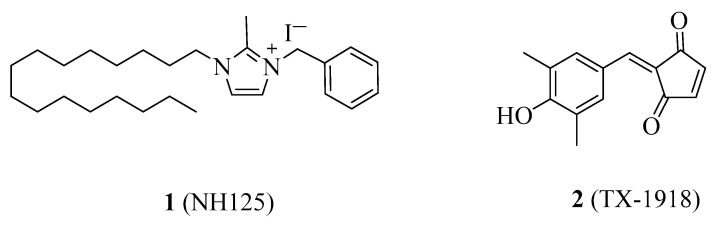
The structures of eEF2K inhibitors **1** and **2**.

NH125, or 1-benzyl-3-cetyl-2-methylimidazolium iodide (**1**, Figure 4), was first discovered to be an imidazolium histidine kinase inhibitor [80]. Later in an eEF2K enzyme activity test on **1** and a series of analogue molecules, this compound was found to significantly inhibit the enzyme activity of eEF2K in vitro (IC_50_ = 60 nM) [81]. The in vitro phenotypic effect of **1** was further tested, and it was found that this molecule inhibited proliferation of several different types of cancer cells [82]. Interestingly, in tumor cells with eEF2K at knocked down levels, compound **1** was still anti-proliferative. In-depth studies have found that the observed effects of **1** in vitro may correlate to induced phosphorylation of eEF2 [82]. Re-examination of the experiment of the inhibition of eEF2K enzyme activity by **1** in vitro led to the verification of this conclusion [83]. In addition, in vivo studies have shown that **1** combined with radiotherapy was more effective than **1** as a single agent or radiotherapy alone in delaying the growth of esophageal squamous cell carcinoma [73].

#### 4.1.2. TX-1918

**Scheme 1:**
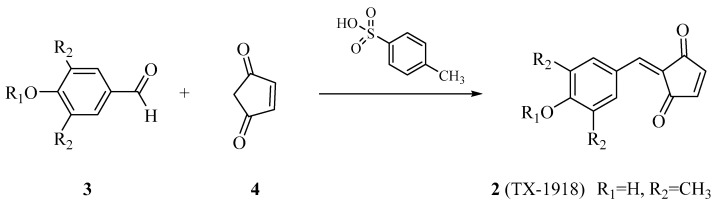
General synthesis of **2** and related analogues.

TX-1918, 2-((3,5-dimethyl-4-hydroxyphenyl)methylene)-4-cyclopentene-1,3-dione (**2**, Figure 4), was discovered as an eEF2K inhibitor with IC_50_ of 0.44 μM in vitro [84]. A series of 2-hydroxyarylidene-4-cyclopentene-1,3-diones was synthesized for testing as protein tyrosine kinase (PTK) inhibitors using a modified Knoevenagel reaction of *m*,*m*′-disubstituted *p*-hydroxybenzaldehydes (**3**, Scheme 1) with 4-cyclopentene-1,3-dione (**4**, Scheme 1) under acidic conditions. It was found that **2** can inhibit various tyrosine kinases, for instance, protein kinase A (PKA): IC_50_ = 44 μM; protein kinase C (PKC): IC_50_ = 44 μM; Src-K: IC_50_ = 4.4 μM; and EGFR-K: IC_50_ = 440 μM, but the necessary concentration is orders of magnitude higher than for eEF2K (IC_50_ = 0.44 μM) [84]. Cytotoxicity testing showed that TX-1918 inhibited the proliferation of HepG2 human liver cancer cells (IC_50_ = 2.7 μM) two orders of magnitude more potently than HCT116 human colon cancer cells (IC_50_ = 230 μM) [84]. This finding emphasizes the important role of the cancer cell type being studied and the related expression level of eEF2K when considering the testing and advancement of eEF2K inhibitors through preclinical development from proteins to cells, and animals. However, the 2-substituted 4-cyclopentene-1,3-dione moiety present in **2** and all analogues prepared by the synthesis shown in Scheme 1 should caution concern of reactivity or toxicity that likely precludes the further development of this series.

While the non-ATP-competitive compounds **1** and **2** have strong inhibitory effects on eEF2K in vitro, their demonstrated anti-proliferative effects at the cellular level are limited, and no in vivo validation has been reported to date. A much greater relative abundance of discovery and development research has focused on ATP-competitive inhibitors of eEF2K.

#### 4.1.3. A-484954 and Its Derivatives

**Scheme 2:**
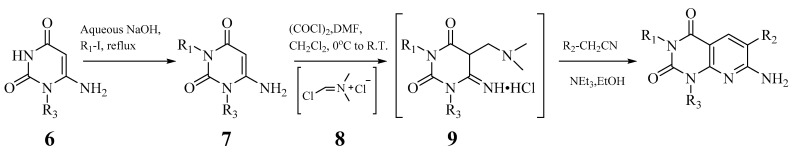
General synthesis of pyrido[2,3-*d*]pyrimidine-2,4-dione analogues.

**Figure 5:**
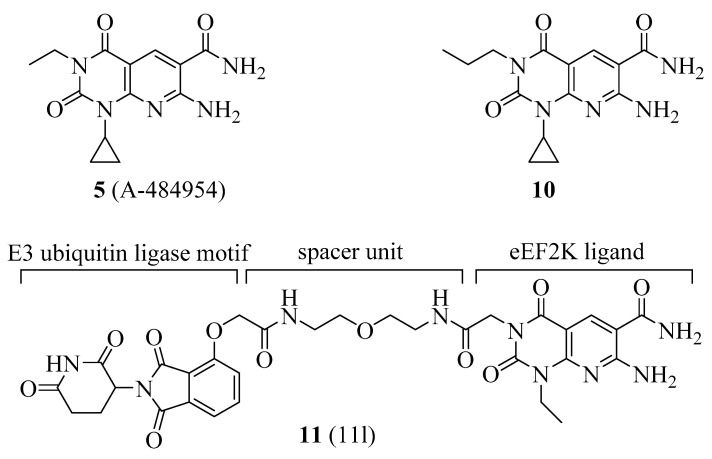
The structures of active pyrido[2,3-*d*]pyrimidine-2,4-diones **5**, **10**, and the related PROTAC lead, **11**.

A-484954, systematically 7-amino-1-cyclopropyl-3-ethyl-2,4-dioxo-1,2,3,4-tetrahydropyrido[2,3-d]pyrimidine-6-carboxamide (**5**, Figure 5), is a reportedly highly selective eEF2K inhibitor with the in vitro IC_50_ value of 0.28 μM that was identified from a chemical library high-throughput screening (HTS) effort using a wide panel of serine/threonine and tyrosine kinases [82]. However, the effect that **5** has on tumor cell proliferation is not obvious until higher concentrations between 10–100 μM and an absence of serum in the bioassay [82,85]. This suggests that the compound may not reach the molecular target in the cell for any of a number of possible reasons. The further design and synthesis of a series of pyrido[2,3-d]pyrimidine-2,4-dione analogues related to **5** have been reported [86]. First, direct alkylation of uracil derivatives (**6**) with alkyl iodides in the presence of aqueous sodium hydroxide afforded the corresponding 1,3-disubstituted-6-aminouracils (**7**) [86]. Next, the hydrochloride (**9**) was prepared by the reaction of the Vilsmeier reagent (**8**) with intermediate **7**, then treated with triethylamine and cyanoacetamide to obtain **5** and a series of analogues in 47–81% yield [86]. As shown in shown in Scheme 2, this synthesis allowed for the rapid generation of analogues but constrained the positions of differentiation available for evaluating a structure–activity relationship (SAR).

The SAR study of pyrido[2,3-d]pyrimidine-2,4-diones indicated that the main pyridine ring and the amido substituent at R_2_ were the key features of this class of molecules that had important influence on the improvement of eEF2K inhibitory activity. In addition, the molecules having the R_1_ group on the scaffold be ethyl (**5**, A-484954) or n-propyl (**10**, Figure 5, IC_50_ = 930 nM) had the most potent inhibitory activity [86]. However, when the R_1_ group was replaced by hydrogen, methyl, or benzyl derivatives, the activity was significantly decreased more than ten-fold [86]. Additionally, moderate loss of activity was observed after changing the cyclopropyl substituent at R_3_ group to ethyl group [86]. Interestingly, when R_3_ was a methyl group, the inhibitory activity was completely abolished [86]. Further study found that analogues **5** and **10** were able to inhibit AMPK-mediated activation of eEF2K [86]. The preliminary SAR study on this scaffold offers insights for the design of new eEF2K inhibitors. In 2020, Liang and coworkers incorporated the “PROteolysis TArget Chimeric” (PROTAC) strategy in combination with lead molecule **5** [87]. The PROTAC strategy involves the recruitment of E3 ubiquitin ligases with a common binding motif that can be tethered across a linker unit to a protein target ligand, here starting with **5** as a moiety designed to target eEF2K. Among a series of synthesized analogues with linkers of various chain length and constitution, compound **11** (“11l”, Figure 5) had the maximum eEF2K degradation rate (Dr) value of 56.7% and could induce apoptosis in human breast carcinoma MDA-MB-231 cells in vitro [87].

#### 4.1.4. TS-2 and TS-4

**Scheme 3:**
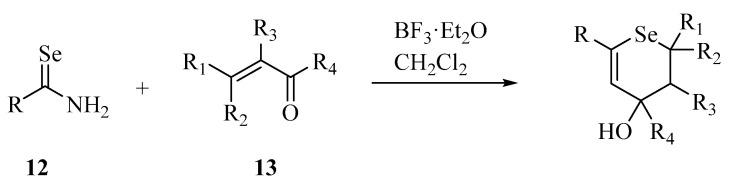
General synthesis of 5,6-dihydro-4H-1,3-selenazine analogues (TS series).

**Figure 6:**
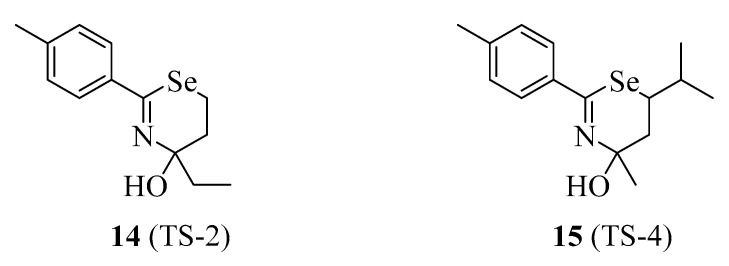
Structures of the active and relatively stable 5,6-dihydro-4H-1,3-selenazines, **14** and **15**.

Ishihara et al. described the synthesis of a series of novel 5,6-dihydro-4H-1,3-selenazine analogues that were prepared by the reaction of selenamides (**12**) with α,β-unsaturated ketones (**13**) under mild conditions in good yields (Scheme 3) [88]. Shortly thereafter, these compounds were screened for inhibitory activity in multiple protein kinases, including eEF2K, PKA, PKC, and protein tyrosine kinase (PTK), CaMK-I/II, and *v-src* [89]. Through this SAR study, 4-ethyl-4-hydroxy-2-p-tolyl-5,6-dihydro-4H-1,3-selenazine (TS-2, **14**, Figure 6) and 4-hydroxy-6-isopropyl-4-methyl-2-p-tolyl-5,6-dihydro-4H-1,3-selenazine (TS-4, **15**, Figure 6) were found to have the most selective inhibitory activity against eEF2K over other protein kinases [89]. The inhibition of eEF2K by **14** and **15** were quantified in a separate purified kinase in vitro, resulting in respective IC_50_ values of 0.36 and 0.31 μM [89]. Compound **14** was further studied and suggested to be an ATP-binding site inhibitor, since it competitively prevented ATP binding to eEF2K and non-competitively blocked eEF2 binding with eEF2K [89]. Further SAR data indicated that when the selenium atom in **14** and **15** was replaced with sulfur, bulky phenyl or styryl substituents were added meta to the toluyl group, or ring size was reduced to 1,3-selenazole, the inhibitory effect for all protein kinases studied was abolished [89]. Although most selenium-containing compounds are relatively unstable, the intramolecular conjugated system comprising the toluyl group and dihydro-1,3-selenazine ring appears to greatly improve stability in this series and offer increased value for these compounds as leads. However, due to these demands and the already explored SAR presented in Scheme 3, there may be limited avenues for further optimization of this series of compounds.

#### 4.1.5. Thieno[2,3-*b*]pyridines

**Figure 7:**
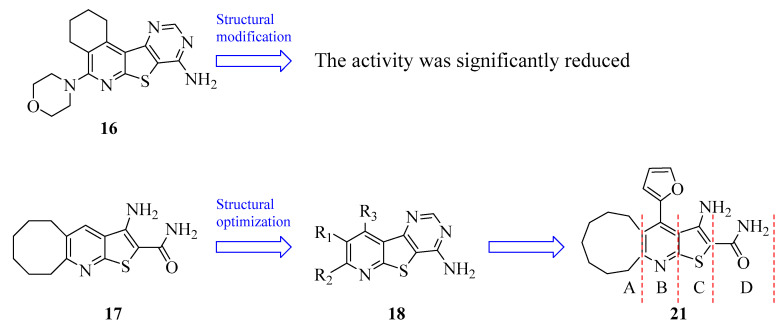
The structures of thieno[2,3-*b*]pyridine analogues.

**Scheme 4:**
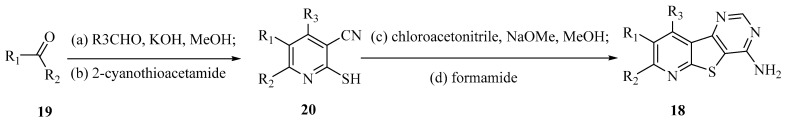
General synthesis of thieno[2,3-*b*]pyridine analogues.

Thieno[2,3-*b*]pyridines were early considered as anticancer agents that target phospholipase C-γ (PLC-γ), and some of these inhibit the proliferation of several breast cancer cell types (MCF7, TamR7, SKBr3, MDA-MB-231/468, etc.) and arrest cells at the G2/M phase [90,91]. Two thieno[2,3-*b*]pyridine-containing heterocycles compounds (**16** and **17**, Figure 7) have been identified as ATP-competitive inhibitors of eEF2K via high-throughput screening, and their IC_50_ values were 0.22 and 2.5 μM, respectively [92]. In a follow-up study, it was determined that even small changes made to the structure of **16** significantly reduced the inhibitory activity against eEF2K, so another structural analogue (**17**) was selected for further optimization [93]. This led to the design and synthesis of a series of thieno[2,3-*b*]pyridine analogues **18** (Figure 7) to be tested as eEF2K inhibitors. The synthetic route (Scheme 4) is briefly described as follows: ketones **19** were reacted with aldehydes in the presence of KOH to generate α-β-unsaturated ketones, which were then cyclized with 2-cyanothioacetamide to obtain the 2-mercaptopyridine intermediates **20** that yielded thieno[2,3-*b*]pyridine analogues **18** after reaction with chloroacetonitrile followed by condensation with formamide [93]. These compounds were subsequently tested for eEF2K inhibition in vitro.

The SAR study of related compounds indicated that position R_3_ on **18** (the B-region indicated on **21**) is open to modification with a wide range of alkyl and aryl substituents while maintaining or moderately enhancing the in vitro eEF2K inhibitory activity; but heterocyclic substitution was preferred [93]. In addition, a six-membered to 10-membered ring size study incorporating the R_1_ and R_2_ positions on **18** (the A-region indicated on **21**) showed the impact on inhibitory activity. For example, incorporating the furan-2-yl group at R_3_, and a nine-membered ring including R_1_ and R_2_, compound **21** (Figure 7) yielded the best activity (IC_50_ = 0.17 μM), followed by the eight-membered (IC_50_ = 0.64 μM) and seven-membered (IC_50_ = 1.1 μM) ring analogues [93]. However, compounds containing six- and 10-membered rings lost activity (IC_50_ > 20 μM) [93]. Among these compounds and the 43 total analogues tested in the same study, **21** demonstrated the highest anti-proliferative activity with EC_50_ of 17 μM against human colon cancer HCT-116 cells [93].

#### 4.1.6. β-Phenylalanines

**Figure 8:**
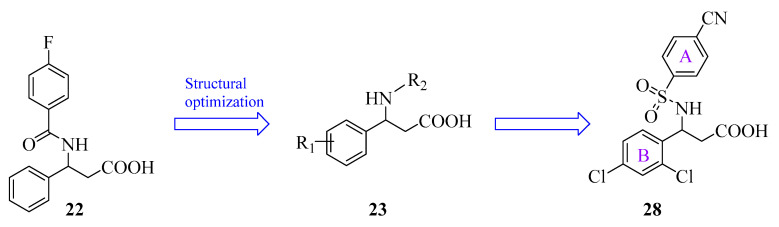
The structures of active β-phenylalanine derivatives and analogues, including **22** and **28**.

**Scheme 5:**
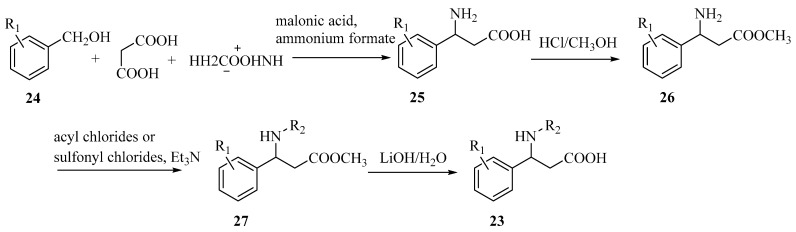
General synthesis of β-phenylalanine analogues.

In 2018, Liu et al. predicted by in silico high-throughput screening a simple β-phenylalanine derivative (**22**, Figure 8) that was later confirmed to have moderate in vitro inhibitory activity on eEF2K (IC_50_ = 35.1 μM) [94]. To evaluate the potential of β-phenylalanine derivatives on eEF2K, a series of 46 such compounds were designed and synthesized (**23**, Figure 8) for evaluation of inhibitory activity on eEF2K and MDA-MB-231/436 breast cancer cells in vitro [94]. The synthetic route to obtain the desired β-phenylalanine derivatives (**23**) is shown in Scheme 5. This uses affordable commercial benzaldehydes **24** and malonic acid as the starting materials to connect by the Knoevenagel reaction to generate substituted β-phenylalanines (**25**). These molecules are simply esterified to afford carboxyl-protected intermediates (**26**) for regiospecific acylation (**27**), and then hydrolysis of the protecting group to yield the desired products (**23**). Overall, this scheme allows for the efficient and affordable generation of a large library of molecules for further testing.

The SAR study on 46 synthesized β-phenylalanine derivatives and analogues indicated that compounds containing the sulfamide group as a linker to a substituted phenyl group with para electron-withdrawing groups, such as CN and CF_3_, showed more potent activity [94]. Introduction of *o,p*-dichloro substitution on the on the β-phenylalanine also increased activity, and 3-((4-cyanophenyl)sulfonamido)-3-(2,4-dichlorophenyl) propanoic acid (“21l”, **28**, Figure 8) yielded the best eEF2k enzymatic activity of compounds tested in vitro (IC_50_ = 5.5 μM) [94]. Molecular docking and molecular dynamic simulations suggest that this molecule is a potential ATP-competing inhibitor. Compound **28** was further found to be weakly anti-proliferative in vitro against MDA-MB-231 cells and MDA-MB-436 cells with IC_50_ values of 12.6 and 19.8 μM, respectively, and most notably demonstrated in vivo efficacy inhibiting tumor growth by inducing apoptosis via eEF2K inhibition in the xenograft mouse model of TNBC with the same two cell lines [94]. The exciting validation of **28** in vivo is promising for the development of this as a lead compound for new anticancer drugs, as well as encouraging for studies of other new ATP-competitive eEF2K inhibitors.

#### 4.1.7. Fluoxetine

**Figure 9:**
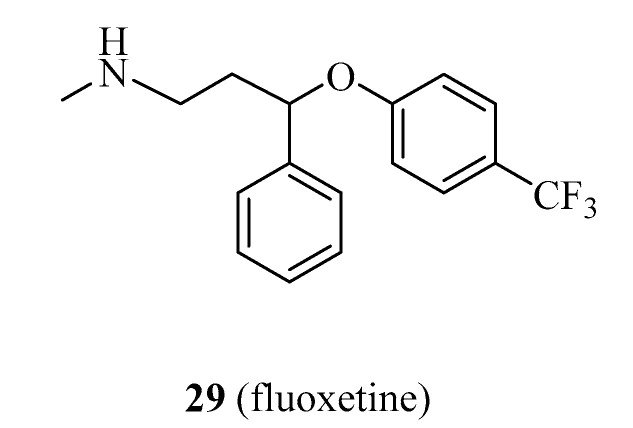
The structure of fluoxetine, an approved SSRI drug later evaluated as an eEF2K inhibitor.

Fluoxetine (**29**, Figure 9) is a selective serotonin reuptake inhibitor (SSRI) drug in widespread clinical use as an anti-depressant under the common trade name “Prozac”. Following a growing trend of drug repurposing and repositioning, **29** has been studied in other systems such as triple negative breast cancer (TNBC). Early evidence has shown that eEF2K is overexpressed in several types of malignancies, especially including TNBC. It was determined that **29** exhibits low-to-sub μM anti-proliferative IC_50_ activities against various TNBC cells in vitro, including MDA-MB-231 and MDA-MB-436 cells [95]. Additionally, **29** induced apoptosis and autophagic cell death in these TNBC cells [95]. The associated anti-TNBC mechanism of action studies has shown that fluoxetine induced autophagic cell death by inhibiting eEF2K and activating the AMPK-mTOR-ULK signaling pathway [95]. These results do well to suggest that inhibition of eEF2K may be a promising treatment strategy for TNBC, and also that other existing drugs may be able to be repurposed or repositioned for eEF2K inhibition. Although the activity of **29** has yet to be proven in vivo for TNBC, the information available about absorption, distribution, metabolism, excretion, and toxicity (ADMETox) of this drug in humans make it an interesting lead for further investigation.

#### 4.1.8. Rottlerin

**Figure 10:**
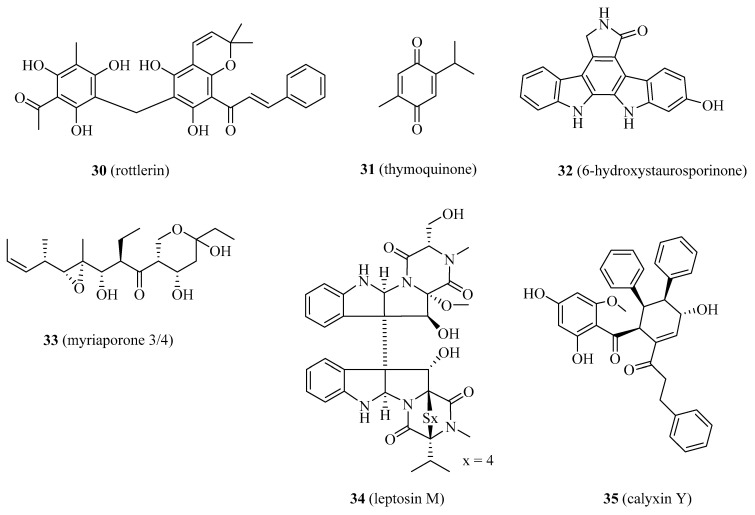
Structures of some natural products that are eEF2K inhibitors (**30**–**35**).

The natural product rottlerin, 5,7-dihydroxy-2,2-dimethyl-6-(2,4,6-trihydroxy-3-methyl-5-acetylbenzyl)-8-cinnamoyl-1,2-chromene (**30**, Figure 10), which is isolated from the pericarps of the red kamala tree *Mallotus philippinensis*, has been used as a protein kinase Cδ (PKCδ) selective inhibitor [96]. This molecule is associated with impacting a variety of cellular processes, such as proliferation, survival, apoptosis, and autophagy [97]. Early studies found that **30** can induce apoptosis on a variety of cancer cell types, such as breast cancer, colon cancer, lung cancer, leukemia, and myeloma [98,99,100,101]. It was initially speculated that this cytotoxic activity of **30** is related to its inhibitory effect on PKC, but it was later found that **30** induces tumor cell apoptosis through inhibition of eEF2K by using reverse phase-protein array (RPPA), cell viability (MTS) assay, and eEF2K knockdown technology [14,75,102]. Several studies have demonstrated that **30** also activates calcium channels and affects the function of mitochondria, further enhancing its use potential as an inhibitor of eEF2K [103,104]. It was reported that **30** significantly inhibits eEF2K at concentrations that are relevant both in vitro and in vivo, and this could explain its antiproliferative activity on human glioma cells and blockage of gliomal cells at the G1–S interface [105]. Furthermore, **30** downregulates the mRNA expression of eEF2K through the ubiquitin-proteasome pathway [105]. Although inhibition of eEF2K is not the only mechanism of action for **30**, this does appear to be one important pathway for its activity profile, which should encourage further studies of structurally related natural products and analogues. Finally, the reported efficacy of **30** for reducing pancreatic tumors in vivo without overt effects on normal tissue offers more validation for both this compound and eEF2K as a molecular target for future drug development [106].

#### 4.1.9. Thymoquinone

Thymoquinone (TQ, **31**, Figure 10) is an active principal natural product found in seeds of the black cumin or Roman coriander plant, *Nigella sativa* [107]. As a potential anti-tumor agent, **31** has been shown to inhibit breast, lung, ovarian, liver, prostate, colorectal, and leukemic cancers in vitro and in vivo [108,109,110,111,112]. Also sometimes considered as a pan-assay interference nuisance compound (PAIN), **31** can interact with a variety of tumor-related targets and pathways, such as nuclear factor-kappa B (NF-κB), tumor necrosis factor-α (TNF-α), STAT3, PTEN, Bcl-2, p53, and PPAR-γ, etc., to exert anti-proliferation, induction of apoptosis and oxidative stress, cell cycle arrest, anti-angiogenesis, and cellular metastasis [109,112,113,114]. After more in-depth research on **31**, it was found that this molecule can also inhibit TNBC cell proliferation and migration/invasion by inhibiting the NF-κB/miR-603/eEF2K pathway [115]. However, there are many obstacles to consider for the development of para-quinones and other PAINs like **31** as drugs and lead compounds, particularly including toxicity.

#### 4.1.10. 6-hydroxystaurosporinone

6-hydroxystaurosporinone (**32**, Figure 10), a bisindole alkaloid, was first obtained from the fruit bodies of Groening’s slime mould, *Lycogala epidendrum* [116]. This compound exhibited in vitro antiproliferative activity against HeLa and Jurkat cells with IC_50_ values of 5.4 and 1.34 μg/mL, respectively [116]. Further in vitro mechanistic studies showed that **32** decreased phosphorylation of protein targets of PKC at 1 μg/mL, along with each eEF2K, PKA, and VEGFR-1 kinase at 10 μg/mL [116]. Other staurosporine analogues have been reported as kinase inhibitors and cytotoxic agents in the past, but these have yet to be tested for inhibitory activity against eEF2K [117]. Further studies could thus provide preliminary SAR data for this series of molecules.

#### 4.1.11. Myriaporone 3/4

The polyketide myriaporone 3/4 (**33**, Figure 10), which was first isolated from the bryozoan false coral, *Myriapora truncata*, has been reported as an inhibitor of eukaryotic protein synthesis [118]. Furthermore, a mechanistic investigation was able to determine that **33** blocks protein synthesis in the elongation stage by directly binding to eEF2K to induce phosphorylation of eEF2 [119]. Compound **33** displays low nM anti-proliferative activity against several cancer cell lines (L-929, PtK2, KB-3-1, PC-3, and A-549) in vitro, inhibits angiogenesis-like tube formation by endothelial cells in vitro in nanomolar concentrations, and is significantly selective (≥ 300x) for acting on cancer cells over normal ones [119]. The potency and selectivity of **33** could make this compound an attractive lead molecule or drug candidate, but the activity apparently has yet to be validated in vivo.

#### 4.1.12. Leptosin M

Leptosin M (**34**, Figure 10) was isolated from a *Leptosphaeria* sp. fungus strain originally separated from growth of the marine alga *Sargassum tortile* [120]. In vitro assays showed that **34** is broadly cytotoxic against a panel array of 39 different human cancer cell lines in low μM concentrations [120]. In addition, **34** was found to inhibit PTK and eEF2K function by 40–70% at 10 μg/mL (~13 μM) but showed no activity against PKA, PKC, or EGFR at 100 μg/mL (~130 μM) [120]. It would thus be interesting to see future studies evaluate the eEF2K inhibitory potential of other natural leptosins or analogues thereof.

#### 4.1.13. Calyxin Y

Calyxin Y (**35**, Figure 10), isolated from *Alpinia katsumadai*, a ginger or galangal used in Traditional Chinese medicine (TCM), exhibits potent antiproliferative activity in several cancer cell lines. One of the many cancer types that eEF2K is overexpressed in is hepatocellular carcinoma (HCC). In 2017, it was reported that a combination of **35** and cisplatin could synergistically inhibit cell viability and induce cell death in both the wild type HepG2 and cisplatin-resistant HepG2 cancer cells [121]. Mechanistic studies revealed that **35** down-regulates eEF2K by promoting SCF βTrCP-mediated protein degradation and enhances the anticancer activity of cisplatin in HCC cells via apoptosis and autophagy [121]. It is exciting that the drug–lead combination also showed in vivo efficacy in a cisplatin-resistant HepG2 xenograft trial without overt toxicity [121], offering some promise of this mixture or other imaginable combinations having use in future therapy.

### 4.2. Discovery and Development of Multi-Target Inhibitors of eEF2K

#### 4.2.1. Inhibitors Targeting PLK1/eEF2K

**Figure 11:**
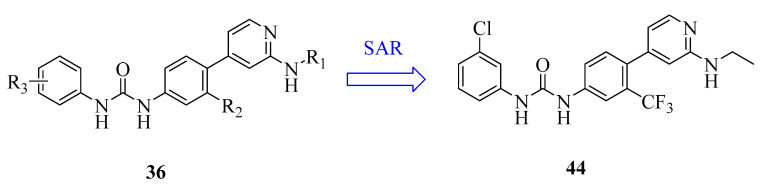
The structures of the PLK1/eEF2K dual-targeting inhibitor, **44**.

**Scheme 6:**
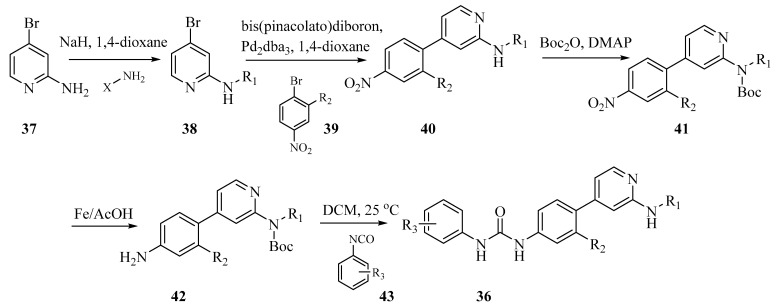
Synthesis route to 1-(4-(2-substituted-pyridin-4-yl)-3-substituted-phenyl)-3-phenylurea PLK1/eEF2K dual-targeting inhibitors.

Multi-target inhibitors are becoming more purposefully developed with the intention of improving efficacy through synergism, safety through reduced dosage, and general prevention of single-target mutation-based drug resistance [127]. A series of 1-(4-(2-substituted-pyridin-4-yl)-3-substituted-phenyl)-3-phenylurea derivatives (**36**, Figure 11) were predicted to be PLK1/eEF2K dual-targeting inhibitors by in silico methods [122]. These compounds were synthesized (Scheme 6), and several were validated as having in vitro inhibition of PLK1 and eEF2K. In brief, 4-bromopyridin-2-amine (**37**) was alkylated to afford **38**, which was then coupled to a series of 2-substituted 1-bromo-4-nitrobenzenes (**39**) via the canonical Suzuki reaction to form intermediates (**40**). After amino protection, these compounds (**41**) were reduced from nitros to amines (**42**), and the final products (**36**) were obtained by N-substitution with the aromatic isocyanates (**43**). The resulting SAR study indicated that the best lead compound was **44** (“18i”; Figure 11), which contains an ethyl group at the R_1_ position, a trifluoromethyl group with the R_2_ position and the 3-chlorine atom at the R_3_ position. This compound showed low micromolar in vitro cytotoxicity against a variety of cancer cell lines and strongly inhibited both target kinases (PLK1 IC_50_ = 0.085 μM and eEF2K IC_50_ = 0.762 μM) [122]. Accordingly, the multi-target strategy appears to be effective with this kinase set and could lead to further exciting developments in the future.

#### 4.2.2. Inhibitors Targeting GLP-1R/eEF2K

**Figure 12:**
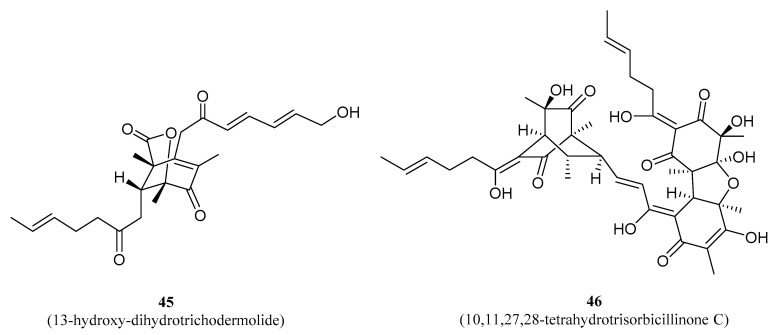
Structures of two active eEF2K inhibiting sorbicillinoid natural products (**45** and **46**) from the sponge-derived fungus *Penicillium chrysogenum*.

In 2019, two new sorbicillinoids, 13-hydroxy-dihydrotrichodermolide (**45**, Figure 12) and 10,11,27,28-tetrahydrotrisorbicillinone C (**46**, Figure 12), were discovered from the sponge-derived fungus *Penicillium chrysogenum* [123]. These natural products strongly inhibit eEF2K and GLP-1R, with Kd values of 118 and 28.5 nM, respectively, for **45**, and 74.6 and 16.2  nM, respectively, for **46** [123]. GLP-1R is more typically related to diabetes than cancers, but it also has implications for various cancers that especially include those effecting the pancreas [128]. The further evaluation of these complex natural products or related analogues in vitro and in vivo could prove to be helpful for the development of eEF2K inhibiting anticancer agents, perhaps especially for pancreatic cancers.

#### 4.2.3. Inhibitors Targeting the Protein/Protein Interaction of Hsp90 and eEF2K

**Figure 13:**
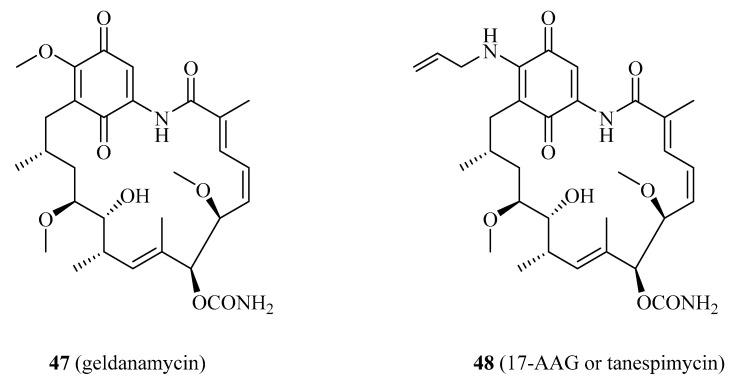
The structures of geldanamycin (**47**) and a synthetic derivative, 17-AAG (**48**).

In 2001, Hait et al. first revealed that the natural product geldanamycin (**47**, Figure 13) and its synthetic derivative 17-allylamino-17-demethoxygeldanamycin (17-AAG or tanespimycin; **48**, Figure 13) had nanomolar inhibitory activity against human glioma in vitro, and significantly inhibited glioma xenografts in nude mice in vivo studies [124]. Further studies showed that these molecules disrupt the interaction of eEF2K with Hsp90 and that this is an important mechanism of cancer cell cytotoxicity for **47** and **48** [124]. At the end of 2020, the Takahashi group reported, after high-throughput screening of a compound library of natural products, that **47, 48,** and related derivatives may inhibit receptor tyrosine kinase-like orphan receptor 1 (ROR1) [125]. This indicates that the inhibition of HSP90 by compounds with the core scaffold of **47** may be promising for treatment of ROR1-positive lung adenocarcinoma, and further expands the known mechanism of action for the molecules [125]. Compound **48** has been the subject of dozens of clinical trials for treating various forms of cancers [126], and the core natural product scaffold of geldanamycin is a promising lead for the development of further generations of inhibitors.

## 5. The Role of Natural Product and Synthetic Small Molecule Activators of Eef2k

**Figure 14:**
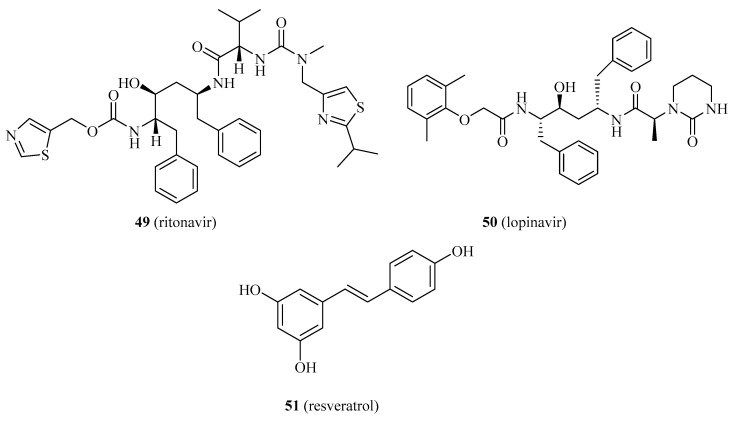
Structures of the eEF2K activators ritonavir, lopinavir, and resveratrol (**49**–**51**).

A continually increasing number of studies have shown that eEF2K is related to the occurrence and development of a variety of tumors, and is considered a potential target for tumor chemotherapy. Subsequently, the development of related inhibitors has also made certain progress. Interestingly, the regulation of tumor autophagy by protein synthesis is bidirectional [129]. Some studies have found that eEF2K activators can also have certain anti-tumor effects. For instance, ritonavir (RTV, **49**; Figure 14) and lopinavir (LPV, **50**; Figure 14) led to an associated increase in eEF2 phosphorylation via the AMPK/eEF2K pathway along with impaired protein synthesis [130,131,132]. Resveratrol (**51**, Figure 14) can inhibit the proliferation and migration of vascular endothelial cells by the activation of AMPK and induced phosphorylation of residue serine 398 of eEF2K, thus leading to inhibited eEF2 activity [133]. The activation of eEF2K by this molecule is illuminating for the multiple directions available for its use as a drug candidate [133]. Huanglian Jiedu decoction (HLJDD), a traditional Chinese Medicinal (TCM), could activate AMPK signaling and further inhibit the mTOR pathway, thus reducing the phosphorylation of eEF2K in hepatocellular carcinoma (HCC) cells [134]. The activated eEF2K leads to the loss of eEF2 activity, and therefore the elongation of new peptides is blocked [134].

## 6. Conclusions and Future Outlook

The compounds here reviewed come from many aspects of medicinal chemistry: computer-assisted drug design, drug repurposing and repositioning, high throughput screening, multi-targeted drug design, natural products discovery and derivatization, and synthetic structure optimization. More than a third of the molecules are natural products that offer complex chemical scaffolds for further optimization, especially after in vivo anticancer activity has been validated (i.e., for compounds **30**, **31**, **35**, **47**, and **48**). However, there are still certain shortcomings to be resolved: (1) Existing inhibitors, and especially the natural products, are not often as selective to eEF2K as would be desired. Therefore, the full mechanism of action of such compounds is not clear. (2) Many current ATP non-competitive eEF2K inhibitors have potent inhibitory effects in vitro, but their anti-tumor effects in vivo have not been studied, are not pronounced, or may be absent for any number of reasons that arise when advancing candidates from protein inhibition assays to whole cells in vitro and onward to animals. (3) While some ATP competitive eEF2K inhibitors show anticancer efficacy in vivo, there remains an opportunity to further optimize their drug-like properties or even potency. (4) No single best core pharmacophore nucleus that specifically inhibits eEF2K has been reported, and the structures of the existing inhibitors are very diverse. (5) The crystal structure of eEF2K remains unknown, and although MHCK A can be used as a template, the similarity between the two is only about 40%. This may contribute to the fact that many designed inhibitors are not yet very specific.

Although important progress has been made in the research and development of eEF2K inhibitors, the complete mechanism of anti-cancer drugs is usually more complicated than single-target action. Further understanding the mechanism of action for the compounds presented here, or the molecular characterization of eEF2K, will help in the optimization of the structure for new eEF2K inhibitors. Additionally, the design and synthesis of purposeful multi-target inhibitors of eEF2K with other relevant anti-cancer targets is also an important research direction for eEF2K inhibitors. Considering the structural diversity and complexity of eEF2K inhibitors, especially natural product inhibitors, how to produce them at scale at low cost remains a problem to be solved. Therefore, further synthetic organic and medicinal chemistry efforts are expected to expand the preclinical and clinical development of eEF2K inhibitors.

Overcoming all of these challenges remains an area of great opportunity for more scientific research efforts to be devoted to the discovery and development of new eEF2K inhibitors. It is suggested that eEF2K inhibitors can be developed as sensitizing agents to improve the outcomes of radiotherapy, as was shown by the in vivo efficacy in an esophageal cancer xenograft of such treatment in combination with compound **1**. Furthermore, since compounds **28** and **30** are effective individual anticancer agents in vivo, **35** in combination with cisplatin can overcome cisplatin-resistance in a HepG2 xenograft, and eEF2K has emerged as one important mechanism for the known single agent anticancer activity of **47** and **48**, this molecular target seems to hold great promise for the development of new first-in-class anticancer drugs.

## Figures and Tables

**Table 1:** Original names and discovery method of reported eEF2K inhibitors.

Compound	Original Name in Published Report	Discovery Method ^a^	Development Status for Cancer Therapy ^b^	Reference
**1**	NH125	KIO	Recent	[73,80,81,82,83]
**2**	TX-1918	KIO	NA	[84]
**5**	A-484954	HTS	Recent	[82,85]
**10**	compound 9	KIO	NA	[86]
**11**	compound 11l	KIO	Recent	[87]
**14**	TS-2	KIO	NA	[88,89]
**15**	TS-4	KIO	NA	[88,89]
**16**	compound 1	HTS	NA	[90,91,92]
**17**	compound 2	HTS	NA	[90,91,92]
**21**	compound 34	KIO	NA	[93]
**22**	compound 9	CADD	Recent	[94]
**28**	compound 21l	CADD	Recent	[94]
**29**	fluoxetine	DRR	Recent	[95]
**30**	rottlerin	NPDD	NA	[96,97,98,99,100,101,102,103,104,105,106]
**31**	thymoquinone	NPDD	Recent	[107,108,109,110,111,112,113,114,115]
**32**	6-hydroxystaurosporinone	NPDD	NA	[116,117]
**33**	myriaporone 3/4	NPDD	NA	[118,119]
**34**	leptosin M	NPDD	NA	[120]
**35**	calyxin Y	NPDD	Recent	[121]
**44**	compound 18i	MTDD	Recent	[122]
**45**	13-hydroxy-dihydro- trichodermolide	NPDD	Recent	[123]
**46**	10,11,27,28-tetrahydro- trisorbicillinone C	NPDD	Recent	[123]
**47**	geldanamycin	NPDD	Recent	[124,125]
**48**	17-AAG	NPDD	Recent	[124,126]

^a^ CADD = Computer-Assisted Drug Design, DRR = Drug Repurposing/Repositioning, HTS = High-Throughput Screening, KIO = Kinase Inhibitor Optimization, MTDD = Multi-Targeted Drug Design, NPDD = Natural Product Discovery and Derivatization; ^b^ Recent = this compound has had literature reports or new developments in cancer research and development published in the past five years, NA = no apparently relevant literature reports or new developments have been found for the same time period.

## Data Availability

Not applicable.
